# Interpreting the Heart-Brain Link: Practical Directions for AI in Cardiac Rehabilitation

**DOI:** 10.34172/jcvtr.33770

**Published:** 2026-06-30

**Authors:** Rehman Bashir, Maheen Imran, Sheza Batool

**Affiliations:** Riphah International University, Islamic International Medical College, Islamabad, Pakistan

## Dear Editor,

 We read with great interest the article by Moreira et al., “Predictive model of neurocognitive functioning after acute coronary syndrome: A machine learning approach.” ^1^ The authors effectively used a Random Forest model in determining the clinical, biochemical, and emotional predictors of neurocognitive outcomes in patients recovering from ACS, an important but often neglected feature of cardiac recovery. This study represents a significant move towards the application of machine learning in integrating neuroscience with cardiology. Yet, before predictive models such as these could be used clinically, a few important factors are yet to be considered.

 First, there is the need for reliability and to minimize overfitting; thus, external validation in larger and more diverse and sex-balanced cohorts is essential. Second, the interpretability of the model is needed. To maintain confidence and clinical applicability, clinicians need to understand how each variable influences the prediction, rather than accept a simple “black-box” result. Explainable AI methods, such as feature importance analysis or SHAP values, may help identify the variables-like HDL, glucose, depression, or BNP-that exert the largest influence on predictions.^2,3^ Finally, neurocognitive changes following ACS result from multiple, multifactorial mechanisms, including mood disorders, perfusion deficits, and inflammatory processes; predictive accuracy should, therefore, be considered within a broader clinical context.^4,5^

 To increase the transparency and robustness of the model, future studies should focus on longitudinal validation to evaluate the Random Forest model’s predictive stability over time by integrating neuro-imaging and inflammatory biomarkers, and assessing alternative algorithms. Integrating these models into digital cardiac rehabilitation platforms may help in early identification of high-risk patients and also allow for customized emotional and neurocognitive therapies that would fit the patients’ needs.

 In conclusion, Moreira et al. have created a promising pathway to link cognitive and cardiac health. AI-driven systems have the potential to develop into dependable clinical instruments that support individualized cardiac rehabilitation and enhance long-term cognitive outcomes with thorough validation and improved explainability.

## Competing Interests

 The author declares no conflict of interest in this study.

## Ethical Approval

 Not applicable.
